# The Second Class of Tetrahydrofolate (THF-II) Riboswitches Recognizes the Tetrahydrofolic Acid Ligand via Local Conformation Changes

**DOI:** 10.3390/ijms23115903

**Published:** 2022-05-25

**Authors:** Minmin Zhang, Guangfeng Liu, Yunlong Zhang, Ting Chen, Shanshan Feng, Rujie Cai, Changrui Lu

**Affiliations:** 1 College of Chemistry, Chemical Engineering and Biotechnology, Donghua University, Shanghai 201620, China; zhangminmin_1950@163.com (M.Z.); zhyl@dhu.edu.cn (Y.Z.); chenting@dhu.edu.cn (T.C.); sandyfss@163.com (S.F.); 2 National Center for Protein Science Shanghai, Shanghai Advanced Research Institute, Chinese Academy of Sciences, Shanghai 201204, China; liuguangfeng@sari.ac.cn; 3 Shanghai Key Laboratory of Plant Molecular Sciences, College of Life Sciences, Shanghai Normal University, Shanghai 200234, China

**Keywords:** noncoding RNAs, riboswitch, tetrahydrofolic acid, conformation switching, THF-II riboswitch, 3D modeling, SAXS, SHAPE, RNA sensors, drug design

## Abstract

Riboswitches are regulatory noncoding RNAs found in bacteria, fungi and plants, that modulate gene expressions through structural changes in response to ligand binding. Understanding how ligands interact with riboswitches in solution can shed light on the molecular mechanisms of this ancient regulators. Previous studies showed that riboswitches undergo global conformation changes in response to ligand binding to relay information. Here, we report conformation switching models of the recently discovered tetrahydrofolic acid-responsive second class of tetrahydrofolate (THF-II) riboswitches in response to ligand binding. Using a combination of selective 2′-hydroxyl acylation, analyzed by primer extension (SHAPE) assay, 3D modeling and small-angle X-ray scattering (SAXS), we found that the ligand specifically recognizes and reshapes the THF-II riboswitch loop regions, but does not affect the stability of the P3 helix. Our results show that the THF-II riboswitch undergoes only local conformation changes in response to ligand binding, rearranging the Loop1-P3-Loop2 region and rotating Loop1 from a ~120° angle to a ~75° angle. This distinct conformation changes suggest a unique regulatory mechanism of the THF-II riboswitch, previously unseen in other riboswitches. Our findings may contribute to the fields of RNA sensors and drug design.

## 1. Introduction

Noncoding RNAs (ncRNAs) have emerged as key regulators in guiding cell proliferation, DNA synthesis or genome rearrangement. They have a variety of biological functions and can regulate gene expression at the levels of RNA processing, transcription or translation [1]. In addition, ncRNAs also serve as crucial biomarkers for human cancer [2,3]. In gene regulatory networks, ncRNAs coordinate other biomolecules participating in multiple biological processes and regulate physiological and developmental processes or even disease [4]. Since the 1990s, the discovery of riboswitches has further propelled ncRNAs research [5,6]. The first riboswitches were found to primarily regulate vitamin B1, B2, and B12 biosynthetic genes, inhibited by thiamine, riboflavin, and cobalamin, respectively [7,8,9]. Shortly after, adenosylcobalamin (AdoCbl) [10], thiamine pyrophosphate (TPP) [11] and flavin mononucleotide (FMN) [12] were proved to bind to mRNAs. Riboswitches are important genetic elements for gene regulation and are located in the 5′ untranslated region (5′UTR) of messenger RNAs (mRNAs) [13]. Unlike many other regulatory elements, riboswitches consist entirely of RNA and can fold into intricate three-dimensional structures [14], responding to small-molecule ligand binding information to regulate gene expression [7]. The selective binding with metabolites, such as coenzymes, derivatives, amino acids or other small-molecule ligands, induces conformational changes and affects transcription termination or translation initiation [15,16,17]. Riboswitches generally contain an aptamer domain and an expression platform [18] and modulate gene expression at various regulatory levels, such as transcription, translation or RNA splicing [12,19,20]. Currently, studies have discovered more than 40 types of riboswitches that recognize more than 30 different small molecules, from ions to vitamins [21]. In general, most bacterial species possess riboswitches, controlling different genes and operons, that account for a significant fraction of their total genetic regulation [7,21].

Recently, new riboswitches have emerged from many bacterial genomes, such as precursors of thiamine biosynthesis (HMP-PP), sixth member of S-adenosylmethionine (SAM-VI) or the second class of tetrahydrofolate (THF-II) [22,23,24]. In this study, we chiefly describe a second class of short ncRNA—the tetrahydrofolate riboswitch (THF-II)—initially called ‘*folE* motif’ RNAs, present in bacterial noncoding RNA domains with riboswitch characteristics [25]. It commonly exists in Gram-negative bacteria, in species of the order Rhizobiales, and associates with genes coding for recombinant GTP cyclohydrolase 1 (FolE) protein, a guanosine triphosphate (GTP) cyclohydrolase enzyme involved in the folate biosynthesis pathway [24,26,27,28,29]. The THF-II riboswitch contains a ~62-nucleotide element, predicted to form an “L” shape with three helical domains connected by loops, and a P3 apical loop [29]. Secondary structure studies showed that the THF-II riboswitch maybe a translationally acting riboswitch, with a Shine–Dalgarno (SD) sequence in the expression platform just upstream of the AUG tail [30,31].

The downstream genes are involved in the transportation and biosynthesis of folic acid and its derivatives [32]. The coenzyme folic acid commonly contributes to the transfer of carbon units in the S-adenosyl methionine structure and to the formation of methyl groups [33]. It plays an important role in the construction of purines and thymidine [34], the metabolism of various nucleic acids and amino acids [35] and carbon metabolism [36,37]. In cells, folic acid is reduced to tetrahydrofolate to carry out its biological functions [38,39]. It carries a carbon unit in the form of methyl, methylene, or formyl, used for the biosynthesis of methionine, thymine, and purine, respectively [40]. In the past half century, antifolates played a pivotal role in cancer therapy [41], targeting folate-dependent mechanisms in humans [42]. Additionally, riboswitches have emerged as molecular sensors [43,44] for developing antibiotics or environmental surveillance, thus warranting extensive studies. In this report, we analyzed the THF-II riboswitch using SHAPE and SAXS, showing its unique conformational and dynamic changes in the presence or absence of a ligand. This paper aims to shed new light on the regulatory mechanism of the THF-II riboswitch.

## 2. Results

### 2.1. Prediction of the Three-Dimensional Structure of THF-II Riboswitch

**Figure 1:**
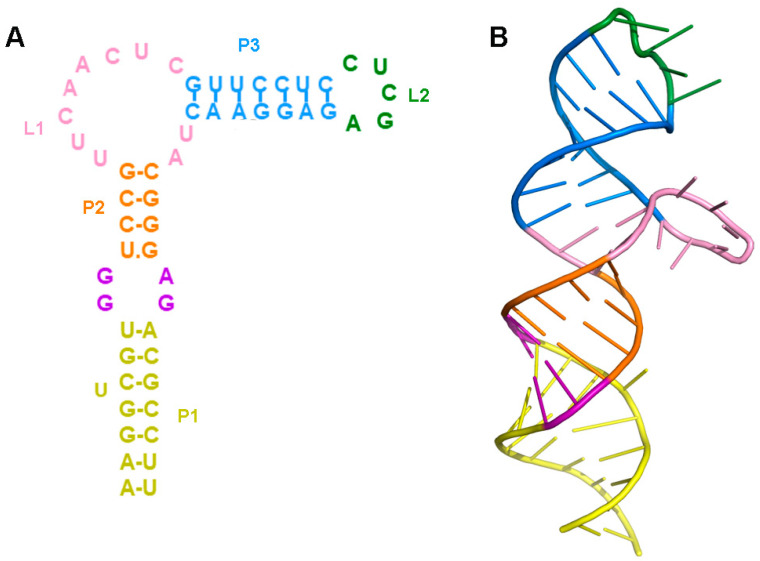
Prediction of the three-dimensional structure of the THF-II riboswitch. (**A**) Sequence and secondary structure prediction of the THF-II riboswitch. (**B**) 3D predicted structure of the THF-II riboswitch. The GGAG-connected P1 and P2 are shown in purple. The Loop1 and Loop2 residues are shown in pink and green. The P1, P2 and P3 stems of the RNA are labeled in dark khaki, orange and blue, respectively.

Since no THF-II riboswitch crystal structure exists to date, we predicted its three-dimensional structure with RNAComposer, an automated RNA structure 3D modeling server. The modeling construct contains 58 nucleotides with three helical domains, P1, P2, P3, held together by loops. Base pairing analyzed in density maps perfectly matched the secondary structure established in previous studies (Figure 1A). The predicted THF-II riboswitch 3D structure shows three dsRNA helices (P1, P2 and P3): P1, P2 are connected by GGAG, and P2, P3 RNA helices are connected by flexible helical turns (Loop1). The helix P3 is capped by Loop2 (Figure 1B), a possible ligand interaction site. A second possible flexible region, Loop1, facilitates a turn on the main helix.

### 2.2. Tetrahydrofolic Acid Recognizes the THF-II Riboswitch via Loop Regions

To investigate the interaction/dynamics between the tetrahydrofolic acid ligand and the THF-II riboswitch, we used selective 2′-hydroxyl acylation analyzed by the primer extension (SHAPE) assay to probe the individual nucleotide flexibility of the THF-II riboswitch.

**Figure 2:**
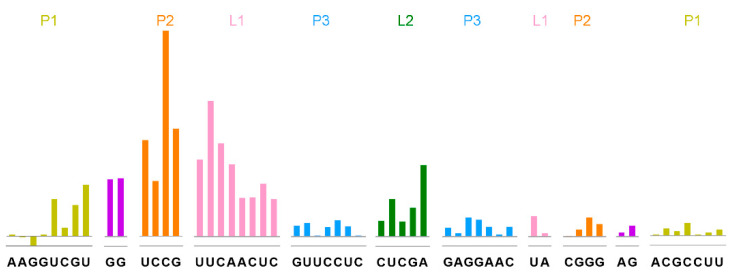
Tetrahydrofolic acid recognizes the THF-II riboswitch. SHAPE analysis showed that tetrahydrofolic acid specifically recognizes the THF-II riboswitch Loop regions. The colored bars represent reduced SHAPE reactivity upon ligand binding. Residues are indicated on the *X*-axis. The coloring of the THF-II riboswitch with SHAPE signal is consistent with the secondary structure in Figure 1. The P1, P2 and P3 stems of the RNA are labeled in dark khaki, orange and blue, respectively. The GGAG-connected P1 and P2 are shown in purple. The Loop1 and Loop2 residues are shown in pink and green.

First, we determined the ligand binding influences on the THF-II riboswitch by investigating both tetrahydrofolic acid-bound and tetrahydrofolic acid-free states. Figure 2 demonstrates the binding pattern differences on the THF-II riboswitch interacting with tetrahydrofolic acid. Overall, ligand binding reduced SHAPE reactivity, as shown by the positive values across the entire RNA (Figure 2). The SHAPE reactivity values for positions in P3 stem (Figure 2, blue bars) showed relatively stable states within the overall structure in both ligand-bound and -free states. Meanwhile, the SD sequence CGGGAGA remained stable (Figure 2, purple and orange bars in the right shoulders of P1 and P2). By contrast, the upstream residues G9, G10 that connect P1 to P2 showed high reactivity in the ligand-free state. Therefore, we suspect that in the absence of a ligand, this sequence might exist as a single strand. Subsequently, the nucleotides in Loop1 and Loop2 (Figure 2, pink and green bars) were unpaired and showed high SHAPE reactivity in the ligand-free state. Our SHAPE results showed that binding with tetrahydrofolic acid did not affect the stability of the P3 helix, but stabilized the local tertiary structure in the Loop regions. L1 and L2 showed high SHAPE reactivity with the ligand, compared with apo-THF. This indicates that tetrahydrofolic acid binding stabilized the loop area of the THF-II riboswitch (Figure 2). Our SHAPE data showed that upon ligand binding, both L1 and L2 significantly lost flexibility (Figure 2, L1, pink bars; L2, green bars). Their drop in SHAPE reactivity exceeded the average signal significantly. This is unequivocal evidence that ligand binding reshaped both loop regions.

### 2.3. Tetrahydrofolic Acid Binding Induces Local Conformational Changes in the THF-II Riboswitch

**Figure 3:**
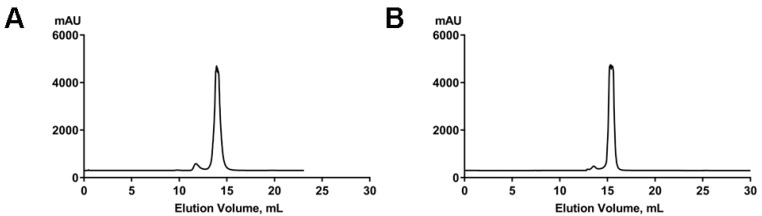
Analysis of the homogeneity and conformation of the THF-II RNA. (**A**) SEC elution profile of the THF-II riboswitch in the absence of the ligand. RNA concentration: 10 mg/mL. (**B**) SEC elution profile of the THF-II riboswitch in the presence of the ligand. RNA concentration: 10 mg/mL. Vertical axes for (**A**,**B**) are in mAU at 254 nm.

To investigate if the THF-II riboswitch underwent global conformational changes upon tetrahydrofolic acid binding, we performed small-angle X-ray scattering (SAXS) on both the ligand-free and the ligand-bound states. The eluted RNAs were characterized by a minor dimerization after size-exclusion chromatography (SEC) (Figure 3). In the absence of the ligand, RNA elution required less than 15 mL, which indicated some aggregation. In the presence of the ligand, the RNA eluted with a slight time delay compared to the apo RNA. This may indicate a reduction in particle size induced by ligand addition. We collected SAXS data using the size-exclusion chromatography-small angle X-ray scattering (SEC–SAXS) method.

**Figure 4:**
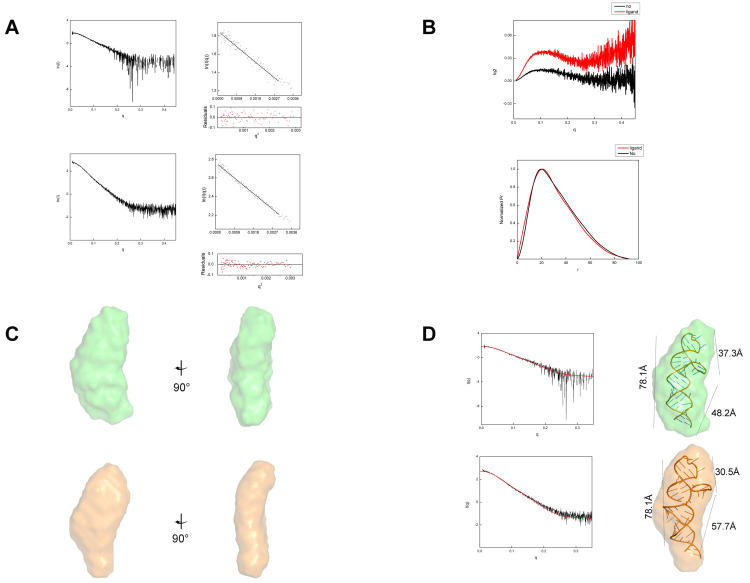
Tetrahydrofolic acid binds to the THF-II riboswitch and induces conformational changes. (**A**) Comparison of experimental scattering profiles (**left**) and Guinier plot (**right**) of the THF-II riboswitch, the ligand-free THF-II riboswitch (upper series) and the ligand-bound THF-II riboswitch (lower series). (**B**) Kratky plot (**upper**) and normalized P(r) analysis (**lower**) of the THF-II riboswitch, the ligand-free THF-II riboswitch (black) and the ligand-bound THF-II riboswitch (red). (**C**) Low-resolution bead models calculated by DAMMIF from the SAXS data. The ligand-free THF-II riboswitch (upper series) is shown in orange, and the ligand-bound THF-II riboswitch is shown in yellow. (**D**) Predicted atomic models of the THF-II riboswitch docked inside the SAXS bead models. The theoretical scattering curve of the THF-II riboswitch predicted atomic structure (red) was compared to the experimental scattering curves (black) by CRYSOL. The coloring of the bead models of the ligand-free THF-II riboswitch (upper series) and ligand-bound THF-II riboswitch (lower series) is consistent with that in panel (**C**). Distances between Loop1 and Loop2, P1 and Loop2 and P1 and Loop1 measure d37.3 Å, 78.1 Å and 48.2 Å, respectively, in the ligand-free state (Figure 4D, upper series), while those in the ligand-bound state measured 30.5 Å, 78.1 Å and 57.7 Å (Figure 4D, lower series).

SEC–SAXS confirmed that the purified THF-II riboswitch particles in the presence or absence of ligand binding were both monodispersed, as shown by chromatography inline X-ray scattering (CHROMIXS) analysis. Ligand-dependent RNA conformational changes can be detected by comparing the scattering profiles. The SAXS profiles of the refolded THF-II riboswitch in the presence or absence of tetrahydrofolate (THF) are shown in Figure 4. Based on SAXS analysis, the Guinier radius of gyration (Rg) for the ligand-free state of the THF-II riboswitch was ∼24.01 Å, only slightly larger than the value of 23.64 Å measured for the ligand-bound state. The paired-distance (P(r)) distributions were calculated from the SAXS profiles (Figure 4A,B and Table 1). The distinct peaks in the Kratky plots of both ligand-free and -bound states of the THF-II riboswitch indicated that the samples were only partially folded in the ligand-free state (Figure 4B).

Our predicted 3D models of the ligand-free state of the THF-II riboswitch (Figure 1A) showed that the overall bead model of the THF-II riboswitch resembled an elongated and twisted letter ‘L’. We then docked the predicted structure of the THF-II riboswitch into our SAXS bead model (Figure 4C). By fitting and linking those structures, we reconstructed 3D predicted models of the ligand-free THF-II riboswitch (Figure 4C, upper series) and ligand-bound THF-II riboswitch (Figure 4C, lower series). The resulting 3D predicted model matched the angles and dimensions of the bead model calculated with SAXS. The CRYSOL results showed that our models of the ligand-free and -bound states of the THF-II riboswitch agreed with the experimental scatter profile (chi^2^ = 1.32 and 2.0, respectively) (Figure 4D). Comparing the 3D bead models of the ligand-free and -bound states of the THF-II riboswitch, we identified two major differences: (i) the ligand-bound bead model appeared compressed between Loop1 and Loop2; (ii) the P2 in the ligand-bound state extended and swung further out of the plane and upwards compared with the apo model. Both changes were attributed only to local conformational changes upon the binding of tetrahydrofolic acid.

Both our SAXS and SHAPE data showed that tetrahydrofolic acid binding compacted and stabilized the THF-II riboswitch. Our fitted 3D model showed that tetrahydrofolic acid binding compressed the loop1 and loop2 (Figure 4D). In the apo THF-II riboswitch, the distances between Loop1 and Loop2, P1 and Loop2 and P1 and Loop1 were 37.3 Å, 78.1 Å and 48.2 Å, respectively (Figure 4D, upper series), while those in the ligand-bound state measured 30.5 Å, 78.1 Å and 57.7 Å (Figure 4D, lower series). Overall, our experiments showed that tetrahydrofolic acid bound to the THF-II riboswitch and altered the conformation and flexibility of the RNA.

### 2.4. Loop1 Rotates in the THF-II Riboswitch upon Ligand Binding

**Figure 5:**
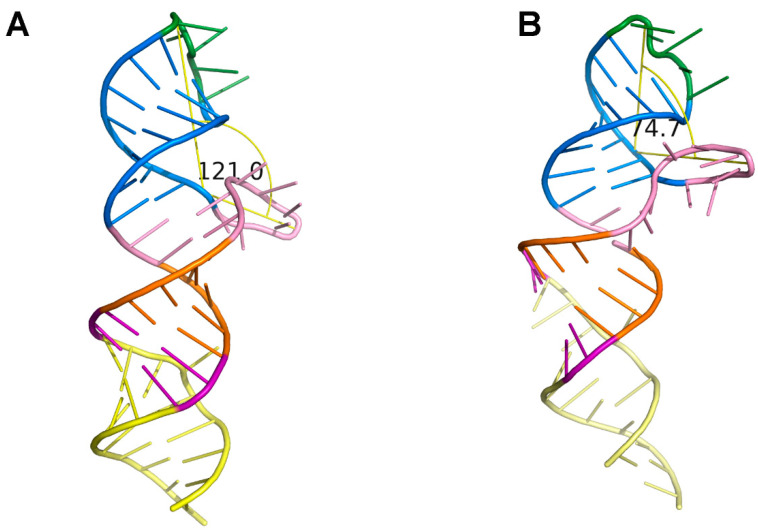
Local rearrangements diagram of ligand binding. (**A**) Angle measurement in the 3D predicted structure in the ligand-free state. (**B**) Angle measurement in the 3D predicted reconstituted structure in the ligand-bound state. The models were predicted with the RNAComposer automated RNA structure 3D modeling server and SHAPE data.

The Kratky plots of these models showed a more defined peak for the ligand-bound state than for the ligand-free model, indicating a more compact ligand-bound model. This observation coincided with our CRYSOL-fitted model that showed the Loop1, P3 and Loop2 loop–stem–loop forming a 75° angle, in contrast with the angle of ~120 ° in the ligand-free model (Figure 5A,B). This positioning of loop-helix elements suggested that Loop1 may contain the tetrahydrofolic acid binding pocket. Due to the fact that the distance between Loop2 and P1 had no change in the +/− ligand model (~78.1 Å), we hypothesized that ligand binding induced conformational changes through local rearrangements in the region Loop1-P3-Loop2.

## 3. Discussion

This study investigated the ligand-free and -bound states of the THF-II riboswitch structure through a 3D structure prediction server, SAXS and chemical probing. Our data show that the THF-II riboswitch bound to tetrahydrofolic acid and existed in a stable conformation in solution. By comparing the SAXS bead models of the ligand-free and -bound states of the THF-II riboswitch, we found that our data support the previous result indicating that tetrahydrofolic acid binds to the loop areas of the riboswitch [29]. According to our SHAPE and SAXS data above, the ligand may bind to Loop1 and Loop2. This result agrees with those previously reported for the tetrahydrofolate (THF) family riboswitch—the first identified class of tetrahydrofolate (THF-I) [32]—but the mechanism of ligand recognition remains unknown.

Despite the lack of a high-resolution THF-II riboswitch tertiary structure, our SAXS data and predicted structural model suggest that the THF-II riboswitch only undergoes local conformational changes upon ligand binding between the Loop1 and the Loop2 regions. THF-II riboswitch has a relatively simple secondary structure compared to the first THF riboswitch, THF-I, which is similar to the SAM-VI riboswitch class [23]. Previous studies showed that ligand binding usually causes global conformation changes, usually involving alternative base paring, as shown by SAXS scattering and/or chemical probing data [45,46,47,48,49]. Generally, apo RNA has usually a larger Rg compared to the ligand-bound RNA. For example, the Rg of the ligand-free TPP riboswitch is 28 ± 1 that decreases to 22 ± 1 in the ligand-bound state [50,51]. Similarly, the Rg of the second member of the S-adenosylmethionine (SAM-II) riboswitch decreases from 21.5 ± 0.2 to 19.5 ± 0.2 upon S-adenosylmethionine (SAM) binding [50]. The purine and lysine riboswitches also undergo substantial conformational changes upon ligand binding [52]. Studies have shown that the effective regulation of gene expression requires high ligand concentrations, ∼100–1000-fold above the dissociation constant (KD), as observed for FMN, the S-adenosylmethionine-1 (SAM-I), and lysine riboswitches [53,54,55]. By contrast, the two states of the THF-II riboswitch showed similar Rg and Dmax (Table 1) in our SAXS data. By comparing the secondary structure and SAXS data of these riboswitches, we speculate that a more complex RNA structure would undergo more noticeable conformational changes upon ligand binding. These global changes usually affect the expression platform by, for example, exposing the SD or forming transcription terminators. The highly flexibility of riboswitches makes crystallization experiments very difficult in the absence of ligands. However, SAXS is an ideal method to obtain the three-dimensional envelope of the apo riboswitch, while SHAPE can pinpoint flexibility changes on individual nucleotides. Through a combination of these techniques, we observed local conformational changes that somehow activated the downstream expression platform, despite the lack of a high-resolution structure and a detailed regulation mechanism. We speculate that this local conformational changes somehow affect the accessibility of the downstream sequence, tipping the equilibrium one way or the other. Previous kinetic studies [47,48,56] showed that smaller riboswitches that do not require alternative base paring are usually involved in translation regulation and offer much faster responses. We speculate that the THF-II riboswitch has similar or better characteristics. Further study is warranted to unveil the function mechanisms of this type of riboswitch.

Furthermore, this study could also benefit the fields of drug discovery and biosensors. Studies have shown that some functional riboswitches can serve as RNA sensors. For example, the tandem glycine aptamer structure from Bacillus subtilis achieved an optimal signal transduction from the sensor to the actuator [57]; metalloriboswitches have evolved ion sensors embedded in the 5′-leader sequences of mRNAs encoding ion uptake or efflux channels [58]; improved riboswitches by RNA amplification appear in the chloroplasts of higher plants [59]. Several established antifolates target folate-dependent mechanisms in humans [42]. Therefore, THF-II ability to bind folates could target this pathway in novel biomedical applications.

Concurrently, riboswitches can be used as targets for antibacterial compounds in drug development and as strain-engineering tools in biotechnology [60]. For example, THF exists in pathogens bacteria of the order *Rhizobiales*, controlling critical cell wall synthesis enzymes in the plant pathogen Agrobacterium tumefaciens synthesis pathways. Targeting these vital biosynthetic pathways can effectively shut down the hypha growth life cycle [61,62,63]. The THF riboswitch also controls folate transport and synthesis in many *Firmicutes* [28]. Developing agonists/antagonists can provide ways to regulate the gut bacterial population, alternative to traditional antibiotics.

Overall, this study established that tetrahydrofolic acid can specifically bind to the THF-II riboswitch, triggering a local conformational switch. The THF-II riboswitch may be a novel target for developing new antibiotics or novel molecular sensors.

## 4. Materials and Methods

### 4.1. RNA Preparation

All RNA plasmids were synthesized by Sangon Biotech, Shanghai, China. They were designed with a T7 polymerase promoter, followed by a 5′linker, the target RNA, a 3′linker and the reverse transcriptase binding site at the 3′ terminus insert into PUC-SP vector. The transcripts were generated by in vitro transcription with T7 RNA polymerase. The RNAs were separated by electrophoresis in a 12% acrylamide denaturing gel; the target RNAs were crushed and eluted into ddH_2_O. The eluted RNAs were buffer-exchanged in Millipore centrifugation columns (3000 Da molecular weight cutoff, Millipore, Germany) to the required concentrations. The RNAs were refolded in a refolding buffer containing 25 mM Tris (pH 7.5), 40 mM NaCl and 10 mM MgCl_2_, heated in a heat block at 95 °C for 3 min, then transferred onto ice for at least 30 min. After refolding, the RNAs were flash-frozen in liquid nitrogen and stored at −80 °C for future use.

### 4.2. Size-Exclusion Chromatography

THF-II RNA in the absence and presence of the ligand as prepared at 10 mg/mL concentration in refolding buffer (40 mM NaCl, 25 mM TriS-HCl pH 7.5, 10 mM MgCl_2_) and run at 0.5 mL/min on a Superdex S200 10/300 GL (GE Healthcare, North Richland Hills, TX, USA) column pre-equilibrated with binding buffer. UV absorbance data were collected at 280 nm and 254 nm.

### 4.3. Small-Angle X-ray Scattering Experiments and Data Analysis

Small-angle X-ray scattering data were collected at the BL19U2 beamline at National Facility for Protein Science Shanghai (NCPSS) and Shanghai Synchrotron Radiation Facility (SSRF). SEC–SAXS was performed on a Superdex 200 Increase 10/300 GL column, equilibrated with 25 mM TriS-HCl (pH 7.5), 40 mM NaCl, 10 mM MgCl_2_. Then, 100 µL of 5 mg/mL samples were injected into the column. SAXS frames were collected at a sample-to-detector distance of about 2.234 m, and the wavelength was set to 1.033 Å. The 2D scattering images were converted to 1D SAXS curves by the software package BioXTAS RAW 2.1.1 [64]. Sample and buffer areas were automatically or manually selected and subtracted by CHROMIXS (chromatography inline X-ray scattering) [65], then the data were forwarded to PRIMUS [66]. The Guinier analysis of Kratky, Pair distribution functions of the particles P(r) and the maximum sizes Dmax were calculated by the program GNOM [67]. Ab initio reconstructions based on the scattering data to create dummy atom models used by DAMMIF/DAMMIN from the ATSAS 2.8.0 program suite [68,69]. DAMAVER [70] was used to align, average and filter the 10 reconstructions. Finally, the calculated envelope was superimposed to the known cryo-electron microscopy (cryo-EM) structure with SUPCOMB [71] and verified with CRYSOL [72]. All models were finally visualized by PYMOL (http://www.pymol.org accessed on 25 March 2022) [73].

### 4.4. SHAPE Sample Preparation and SHAPE Experiments of THF-II RNA

We prepared four 1.5 mL tubes and added 9 µL of refolded RNA in each. The control with only THF-II and the sample with THF-II plus tetrahydrofolic acid were treated with dimethyl sulfoxide (DMSO); the probing reagent 1M7 was added to the probing reaction mixtures at a final concentration of 10 mM, then reaction was completed in 5 min on ice. Then, RNA was recovered by ethanol precipitation with sodium chloride, glycol-blue and stored for 40 min at −80 °C. After centrifugation, the RNA samples were resuspended in 9 µL of ddH_2_O.

B The 9 µL RNA and two additional 9 µL RNA samples (prepared for 2′,3′-dideoxyadenosine 5′-triphosphate (ddATP, Trilink Biotechnologies, San Diego, California, USA) and 2′,3′-dideoxyguanosine 5′-triphosphate (ddGTP, Sigma-Aldrich, Merck KGaA, Darmstadt, Germany) sequencing) were combined with 1 µL of a FAM-5′end-labeled DNA primer and allowed to anneal at 65 °C for 5 min, followed by incubation at 35 °C for 5 min and cooling to 4 °C. Then, 6 µL of reverse transcription (RT) mix (4 µL of 5 × first-strand buffer,1 µL of 0.1 M DTT, 1 µL of 10 mM dNTPs mixture) was added to the reactions, followed by incubation for 5 min and by incubation at 49 °C for 1 min. Subsequently, we added 1.5 µL of SuperScript II reverse transcriptase (200 U/µL; Invitrogen, Waltham, MA, USA), and further incubated the mixture at 49 °C for 29 min and then at 95 °C, after the addition of 1 µL of 4 M NaOH, for 5 min. The reactions were stopped by the addition of 29 µL of 1 M acid stop dye, incubation at 95 °C for 5 min and cooling to 4 °C. The cDNA fragments were analyzed by short tandem repeats (STR) analysis and quantified using the Shape Finder software.

## Figures and Tables

**Table 1:** Radius of gyration (Rg) and maximum dimension (Dmax) for the THF-II riboswitch in different solution conditions, measured by SAXS.

Structural Parameters	Ligand-Free	Ligand-Bound
I(0) (cm^−1^) from Guinier fit	6.44 ± 0.048	15.95 ± 0.065
Rg (Å) from Guinier fit	24.01 ± 1.21	23.64 ± 1.35
Rg (Å) from P(r)	25.15	25.08
Dmax (Å) from P(r)	89.46	92.5
I(0) (cm^−1^) from P(r)	25.18	25.12

## Data Availability

Not applicable.
